# Open Field Release of Genetically Engineered Sterile Male *Aedes aegypti* in Malaysia

**DOI:** 10.1371/journal.pone.0042771

**Published:** 2012-08-27

**Authors:** Renaud Lacroix, Andrew R. McKemey, Norzahira Raduan, Lim Kwee Wee, Wong Hong Ming, Teoh Guat Ney, Siti Rahidah A.A., Sawaluddin Salman, Selvi Subramaniam, Oreenaiza Nordin, Norhaida Hanum A.T., Chandru Angamuthu, Suria Marlina Mansor, Rosemary S. Lees, Neil Naish, Sarah Scaife, Pam Gray, Geneviève Labbé, Camilla Beech, Derric Nimmo, Luke Alphey, Seshadri S. Vasan, Lee Han Lim, Nazni Wasi A., Shahnaz Murad

**Affiliations:** 1 Oxitec Sendirian Berhad, Kuala Lumpur, Wilayah Persekutuan, Malaysia; 2 Oxitec Limited, Oxford, Oxfordshire, United Kingdom; 3 Medical Entomology Unit, Institute for Medical Research, Kuala Lumpur, Wilayah Persekutuan, Malaysia; 4 Centre for Research in Biotechnology for Agriculture, University of Malaya, Kuala Lumpur, Wilayah Persekutuan, Malaysia; 5 Department of Zoology, University of Oxford, Oxford, Oxfordshire, United Kingdom; University of Crete, Greece

## Abstract

**Background:**

Dengue is the most important mosquito-borne viral disease. In the absence of specific drugs or vaccines, control focuses on suppressing the principal mosquito vector, *Aedes aegypti*, yet current methods have not proven adequate to control the disease. New methods are therefore urgently needed, for example genetics-based sterile-male-release methods. However, this requires that lab-reared, modified mosquitoes be able to survive and disperse adequately in the field.

**Methodology/Principal Findings:**

Adult male mosquitoes were released into an uninhabited forested area of Pahang, Malaysia. Their survival and dispersal was assessed by use of a network of traps. Two strains were used, an engineered ‘genetically sterile’ (OX513A) and a wild-type laboratory strain, to give both absolute and relative data about the performance of the modified mosquitoes. The two strains had similar maximum dispersal distances (220 m), but mean distance travelled of the OX513A strain was lower (52 vs. 100 m). Life expectancy was similar (2.0 vs. 2.2 days). Recapture rates were high for both strains, possibly because of the uninhabited nature of the site.

**Conclusions/Significance:**

After extensive contained studies and regulatory scrutiny, a field release of engineered mosquitoes was safely and successfully conducted in Malaysia. The engineered strain showed similar field longevity to an unmodified counterpart, though in this setting dispersal was reduced relative to the unmodified strain. These data are encouraging for the future testing and implementation of genetic control strategies and will help guide future field use of this and other engineered strains.

## Introduction

Dengue is the most important mosquito-borne viral disease, with an estimated 50 million cases per year and increasing incidence and severity [1], [2]. There are no specific prophylactic or therapeutic drugs for dengue and no licensed vaccine. Control of the vector mosquito is therefore the only way to control or prevent dengue. Current methods are based on the elimination or insecticidal treatment of larval habitats, or ULV spraying (fogging) with insecticides to try to kill adults. However, these methods are of limited effectiveness. Even the most rigorous application has failed to prevent epidemic dengue in endemic areas; for example Singapore still has thousands of cases of dengue each year despite the efforts of its highly organised and well resourced mosquito control programme [3], [4]. New technologies that are able to reduce the vector population below the dengue transmission threshold are therefore required.

One such method is the Sterile Insect Technique (SIT) wherein large numbers of sterile male mosquitoes are released to mate with the wild females, thereby reducing their reproductive potential. This approach is species-specific and environmentally friendly, since it does not involve the use of chemical insecticides. SIT has been successfully used to suppress or eliminate some major agricultural pest species including the New World screwworm (*Cochliomyia hominivorax*) and the Mediterranean fruit fly (*Ceratitis capitata*) [5], [6]. Despite some successful trials [7], [8], the method has not achieved large-scale application against mosquito vectors, in part due to the damaging effect on mosquitoes of sterilising doses of radiation. The possibility of genetics-based enhancements to the SIT potentially allows this approach to become a major new tool and component of integrated vector management [9]–[15]. Key potential benefits include genetic sterility [10], [16] (e.g Release of Insects carrying a Dominant Lethal, RIDL [13], [14]), the potential to select the developmental stage at which lethal traits manifest [13], [15], [17], genetic sexing [14], [18]–[20], the provision of a fluorescent marker to allow the discrimination of transgenic and wild type insects [13], and resistance management for other interventions [21]–[24]. Analysis indicates that this approach is likely to be attractive on cost-effectiveness as well as efficacy grounds relative to existing alternatives [17], [25].

OX513A is a RIDL strain which carries a repressible dominant lethal transgene insertion causing lethality at a late larval or early pupal stage unless reared in the presence of tetracycline [13]. In its proposed operational use, OX513A homozygous males would be released to mate with wild females. Progeny of such mating would inherit one copy of the OX513A insertion and consequently die before adult metamorphosis. Exactly as with classical SIT, if sufficient wild females mate with OX513A males, the target population will diminish.

One potential objection is that the genetic engineering may itself have a strongly negative impact on the performance of the engineered males, thereby reducing the net improvement over radiation-sterilised insects [26]. Such negative effects have been seen in some strains of transgenic insects [27], [28], but not in others [29]–[32]. In particular, OX513A has shown only minor differences in life history and performance traits relative to wild-type laboratory comparator strains in laboratory assays [13], [33]–[37]. Mating competitiveness studies conducted in the lab [33] and in semi-field conditions [38] showed little or no difference in mating capacity between wild-type and OX513A males. However lab and semi-field assays may not accurately predict field performance. The key performance parameters for released males are longevity, dispersal and mating competitiveness. In a previous study in Grand Cayman we found that released OX513A males competed well for mates [39]. Here we describe a field experiment in Malaysia addressing dispersal and longevity of released OX513A males, both in absolute terms and relative to an unmodified wild type laboratory comparator strain.

## Methods

### Regulatory affairs and community engagement

Regulatory permission was sought and received in accordance with the Malaysia Biosafety Act (2007). This process included scrutiny from the Institutional Biosafety Committee (IBC) of the Institute for Medical Research (IMR), Kuala Lumpur, the Ministry of Health's Medical Research Ethics Committee (MREC) and the Genetic Modification Advisory Committee (GMAC) at the Ministry of Natural Resources and Environment (NRE) (http://www.biosafety.nre.gov.my/country_decision/app_ft.shtml). The final approving body was the National Biosafety Board (NBB) of NRE; NBB approved the project on 5 October 2010 [permit no. JBK (S) 602-1/1/3(29)].

As part of an incremental, stepwise approach to testing the strain, the first open field release was to (i) involve a small number of males and (ii) be conducted in an uninhabited area. *Ae. aegypti* is strongly human-associated, so uninhabited areas, including the forested area selected, are not typical habitats. At the time this study was designed, no engineered mosquitoes had been deliberately released anywhere in the world. The study was designed to test the effect of the transgene insertion into the mosquito genome by comparing two similar strains (except for the RIDL insertion); even in an atypical environment the strains can still be compared. The present study was designed to look for significant differences, if any, in dispersal and survival between the two strains. These two parameters are critical in designing suppression programmes, as they will drive the frequency of releases and distance between release points, and also contribute to the risk assessments for subsequent studies. Studies to assess mating capacity in the field were (and still are) planned to be conducted in a larger scale study in typical environments for *Ae. aegypti* in Malaysia.

As part of the NBB's approval process, the intent to conduct limited releases was advertised twice in two national newspapers during August 2010, the Berita Harian (in Bahasa Malaysia) and the New Straits Times (in English), as part of a 30-day public consultation process. In order to proactively solicit comments from non-governmental organisations (NGOs) and improve the risk assessment process, the NRE also wrote to nine NGOs, and requested meetings with NGOs including the World Wide Fund for Nature and Third World Network. Taking into account the information and comments received during this consultation, GMAC concluded that the limited field trial would not endanger biological diversity nor human, animal or plant health. After considering all inputs, comments and concerns, NBB granted approval for IMR's limited open release experiment, with specific terms and conditions, in accordance with the Biosafety Act 2007.

As part of public engagement prior to open release in the uninhabited site, NRE asked IMR to obtain permission from local government authorities, and to display large multi-lingual posters in the uninhabited trial site for at least two weeks prior to the date of release. These conditions were met by IMR to the satisfaction of NRE inspectors at the trial site located in an uninhabited area in Bentong district. Permission was also obtained from Pahang state authorities. In addition, IMR also participated in public meetings arranged by the Bentong Municipal Council and the Bentong Malaysian Chinese Association in which information was presented to the local community using visuals and non-technical language (Bahasa Malaysia and Mandarin). These public meetings reinforced the ground-level support for the trials and positive feedback from the local community was reported by The Star (“GM mosquito plan gets the thumbs-up”, 1 November 2010) following its independent survey of Bentong residents. NRE has carefully observed the entire implementation and progress of the project and details have been made available on the IMR website (http://www.imr.gov.my/component/content/article?id=1119).

After the end of the study, the entire site was treated twice by thermal fogging with Resigen™ (Bayer CropScience AG, Leverkusen, Germany) on the 6^th^ January 2011 and the 18^th^ January 2011 by the Vector-borne Disease Control Programme in the Bentong Public Health district in accordance with the Malaysian Ministry of Health guidelines for dengue control. This was one of the conditions of the trial stipulated by NRE. Both strains used in this study were found to be equally susceptible to a range of insecticides including the pyrethroids used in the study site [37].

### Field site

**Figure 1:**
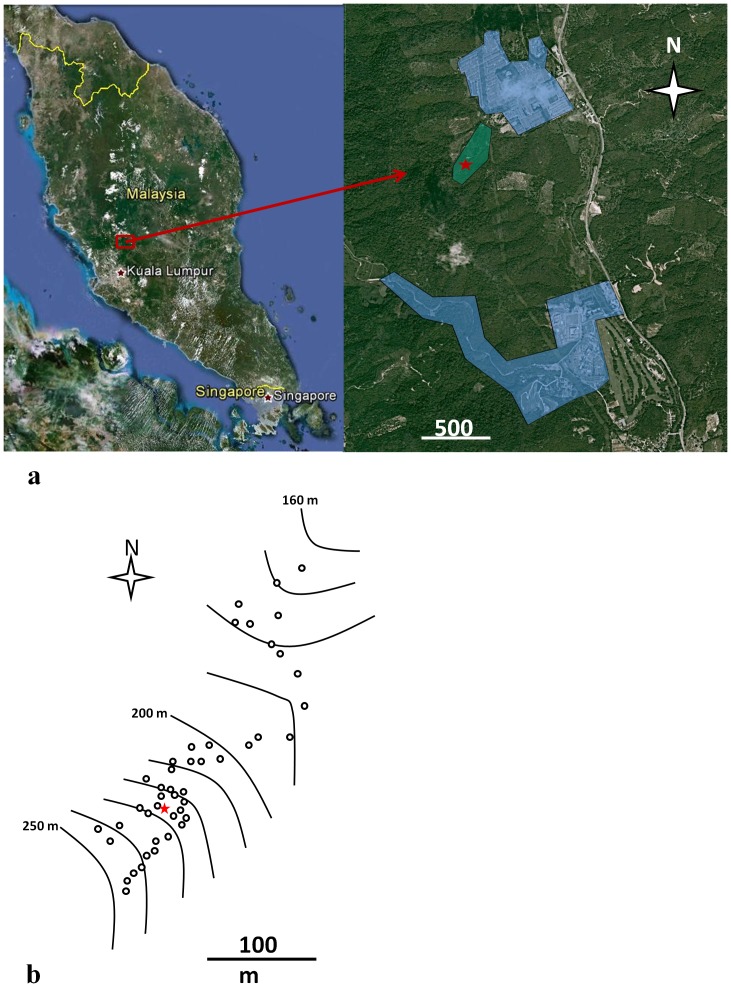
Study and monitored areas in Bentong, Pahang, Malaysia. (a) The release was conducted in an uninhabited area comprising a jungle area (government land), a cleared area and a young rubber plantation where a network of 45 adult traps (BG-Sentinel) and 44 ovitraps were set. The closest inhabited areas were monitored with 35 ovitraps only, (release point: red star; uninhabited study area: green area; inhabited monitored area: blue area) (Credits for small scale map: © 2012 Google; © 2012 Tele Atlas; © 2012 TerraMetrics; Credits for large scale map: © 2012 Google; © 2012 GeoEye; © 2012 Cnes/Spot Image; © 2012 Mapit). (b) The BG-Sentinel traps were principally set in the cleared area on the small terraces surrounding the release point and uphill on a small path through the forest until around 100 m from the release point. Further traps were placed downhill in the rubber plantation along the road leading to the closest inhabited area. (Release point: red star; BG-Sentinel™ traps: circles; Altitude: contour lines (separated by 10 m)).

The release site is located in *Hutan Tanah Kerajaan* (*Bukan Hutan Simpanan* or non-reserved government forested land) off Jalan Tentera, which is off the highway known as “Lebuhraya Bentong-Raub”, Bentong district, Pahang, Malaysia (Figure 1a). It is an uninhabited area on the side of a hill comprising a jungle area (government land), a cleared area and a young rubber plantation (private land). The cleared area is a low bush vegetated area with numerous cut trees and low vegetation cover. Posters announcing that a limited trial with transgenic mosquitoes was being conducted were placed downhill (340 m from release point) and uphill (130 m from release point) 22 days in advance of the release, in accordance with the requirements of the regulatory authorities, and maintained until the end of the study. Prior to release, written informed consent was received from the landowners. The release point (3°33.92′N, 101°52.99′E) was in a cleared area approximately 100 m from the rubber plantation. The nearest inhabited areas were over 500 m to the north-east and over 1 km to the south-east and south-west of the release point (Figure 1a). A weather station was set up in the area to record temperature, humidity, rainfall and wind direction and strength.

### Rearing

Mosquitoes were reared in a dedicated facility (ACL-2) at the IMR at 27.5°C (±1°C) and 70% (±10%) humidity. Eggs were hatched under vacuum. Larvae were reared in trays (23×30.5×8 cm) containing 1 L of water and fed daily with Vipan fish food (Sera®, Heinsberg, Germany). Two strains were used during this experiment, a laboratory strain originating from Jinjang, Kuala Lumpur, Malaysia, that has been reared in the IMR since the 1960s (referred as My1 strain), and an OX513A strain. This OX513A strain was constructed by making a line homozygous for the OX513A insertion after introgressing the insertion from its original Rockefeller strain background [13] into the My1 strain by backcrossing for 5 generations such that ∼97% of the genome of the resulting strain, termed OX513A-My1, is expected to derive from the Jinjang strain. Both strains were reared at the same initial density and feeding protocol.

### Sorting

After pupation, larvae and pupae were separated, first mechanically on the basis of size [40], [41] and then manually by microscopic examination. 6,500 pupae of each strain were allowed to eclose into a cage (38×38×38 cm); emerged adults were provided with 10% sugar solution supplemented with vitamin B complex solution until their release. Additional sorted pupae were re-examined to assess the sorting efficiency; adults were also visually checked for the presence of females the day before their release. Three days after sorting, the pots containing the pupae were removed from the cages to count dead pupae, live pupae and dead adults remaining in the pots.

### Marking and release

Release was conducted four days after pupal sorting when the males were already sexually mature, i.e. 3±1 day-old [42]. On the day of release, cages covered with a wet cloth were put in a secure plastic box and taken to the release point. Cages were transferred to a plastic bag and sprayed with fluorescent powder (Day Glo; Switzer Brothers, Cleveland, OH) following a standardized protocol which had been found not to adversely affect the lifespan or dispersal of wild type mosquitoes in previous laboratory and field assays (McKemey, unpublished data): Saturn Yellow (A-17-N) and Red Rocket (A-13-N) powders for the My1 and OX513A-My1 males, respectively. The release was done using a simple manual rope remote opening to enable the operators to open the upper part of the cage at a 10 m distance; the operators then left the area rapidly to limit potential bias to the males' dispersal caused by their presence. Cages were then left for 15 minutes before being collected and brought back to the lab to count the number of dead and non-released adults.

### Recaptures

A network of 45 BG-Sentinel traps (Biogents™, Regensburg, Germany, http://www.bg-sentinel.com) was set around the release point (Figure 1b). Owing to the topography and vegetation of the site, traps could not be set evenly in every direction from the release point. The furthest traps from the release point were 96 m and 328 m uphill and downhill, respectively. Traps were baited using BG-lure (Biogents™) and their positions recorded with GPS. Traps were powered by sealed batteries (12V, 12Ah, JSB 12120™), which were changed and charged daily. Nets were collected and replaced daily and all trapped mosquitoes taken to the laboratory for identification. The trapping period was from the time of release (Day 0) until three consecutive days without recaptures (Day 15).

The size of a sample of the recaptured mosquitoes was assessed by measuring the distance from the auxiliary incision to the apical margin of the wings, excluding the fringe of scales [43]. Digital images of the wings alongside a micrometer, for purposes of scale, were taken using a Nikon DSFi1 camera and analysed using ImageJ 1.42q (NIH, USA).

### Monitoring

A network of ovitraps was set in the area weekly. Ovitraps were set at least at 5 m from BG-Sentinel traps to minimize interference with the adult traps. The ovitrap sampling described by Lee [44] was used for surveillance, based on the Malaysian Ministry of Health Guidelines [45]. The ovitrap is a 300 ml cylindrical plastic container part-filled with water (approximately 200 ml) within which a hardboard paddle (10×2.5×0.3 cm) is placed with the rough surface upward; this provides an oviposition substrate. Forty four traps were placed in the uninhabited area and 35 were placed in the nearest inhabited places to monitor presence and abundance of wild mosquito populations, as availability of female *Ae. aegypti* could impact dispersal behaviour (Figure 1). In addition the ovitrap can be used to monitor dispersal and persistence of the RIDL gene into the environment by checking the eggs for presence of RIDL gene. Ovitraps were brought back to IMR after one week; recovered larvae were identified by species. First and second instar larvae were scored for fluorescence; all larvae were allowed to develop to adult and then genotyped by PCR.

### PCR

Genomic DNA was extracted from adults, using the GeneJET DNA purification kit (Fermentas) according to the manufacturer's instructions. PCR was carried out using 2 primer pairs, and DreamTaq™ polymerase (Fermentas), using a touchdown PCR programme with annealing temperature decreasing by 0.5°C/cycle over 10 cycles, from 55°C to 50°C then 25 cycles with annealing at 50°C. Primers AeA4F2 (CAATCGAAGCGAGGTATCCTCACCC) and AeA4R2 (CTGGGTACATGGTGGTACCACCAGAC) amplified the *Actin-4* gene, so acted as a control for DNA quality. Primers WT1 (GAAATCCCCTAGTAAAATTCGCGGAGAAATTC) and IRV1 (CGTCATTTTGACTCACGCGGTCGTTATAGTTC) amplified across the insertion-flanking sequence boundary so would only be positive in insects carrying the OX513A transgene. OX513A-My1 gDNA, known to amplify with the WT1-IRV1 primer pair, was also included as a positive control.

### Statistical analysis

Statistical analysis was performed using R (R Core Team, Vienna, Austria). Dispersal of released males was analysed using the Mean Distance Travelled (MDT) method described by Morris which provides corrections for trap density [46]. Flight range 50 and 90, i.e. estimates of the distance from the release point within which 50% and 90% of the released males were recaptured, were also calculated [46]. Survival in the field was estimated using Buonaccorsi's non-linear method [47]; life expectancy was derived from the survival estimate using the formula −1/log_e_(survival) [48]. Confidence intervals for survival and dispersal were calculated by bootstrap (1,000 repeats). As the wing length of non-released OX513A-My1 males was not normally distributed (Kolmogorov-Smirnov: p<0.05), Wilcoxon tests were performed to assess the difference between strains.

## Results

### Quality control of sex sorting

Presence of females after grid sorting was 0.33% (33/10,029) for the OX513A-My1 strain and 12.7% (946/7,446) for the My1 strain. Higher proportional presence of females in the My1 strain was due to low adaptation of My1 strain to mass rearing conditions, resulting in smaller pupae. Thus, while conducting the mechanical sex sorting, more female pupae were able to pass through. Use of a narrower sorter might have eliminated some larger males; since the size of males has been reported to affect field longevity [49] this was considered undesirable, and in any case the remaining females were all removed manually before release. Out of the 21 groups of OX513A-My1 male pupae from the first manual sex-sorting, 6 were manually checked by microscopic examination for a second time. No females were found in these 2,907 pupae which were double-checked. A final pre-release inspection of adults also found no females.

### Weather data

During the release at midday on 21 December 2010, the temperature was 32.6°C with relative humidity of 66%, a wind velocity of 1 m/s and no precipitation. The wind was mainly toward the north (north-east to north-west) with speed not exceeding 15 km/h. Over the study period, the average temperature was 25.8°C (20.4–41.7°C), the average humidity 81.8% (42–95%) and the total rainfall was 114.8 mm which are normal conditions in Malaysia at this time of the year.

### Recaptures

**Figure 2:**
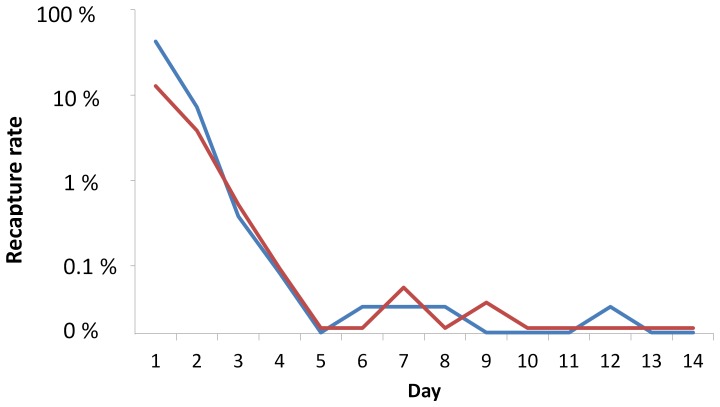
Recapture rate over time of OX513A-My1 and My1 males. Adult traps (BG-Sentinel) were serviced daily; the recapture rate of each released male type caught each day following release on Day 0 is plotted (OX513A-My1: blue; My1: red). Similar recapture rates over time were observed for each strain.

Traps were collected up to 15 days post release from 22 December 2010 to 5 January 2011. Total recapture rates were 50% (3,034/6,045) and 17% (925/5,372) for OX513A-My1 and My1 strains, respectively (Table 1). Trap number 10, situated 13 m from the release point (the closest trap), recaptured 1,258 (41% of total recaptures) and 247 (27% of total recaptures) OX513A-My1 and My1 males, respectively, on day 1 alone (Figure 2).

**Table 1:** Number of males released, recapture rates and total recaptures for OX513A-My1 and My1 laboratory strains.

Strain	Males released	Recapture rate	Recaptures
OX513A-My1	6,045	50.2%	3,034
My1	5,372	17.2%	925

### Wing measurement

A sample of the males recaptured on day 1 (OX513A-My1: 78; My1: 77) and 2 (OX513A-My1 : 77; My1: 43) in each annulus and all the males recaptured after day 3 (OX513A-My1 : 23; My1: 26) were measured in the laboratory (Table 2). One hundred and fifty OX513A-My1 males were also measured in the lab from stocks that had not been released. The recaptured OX513A-My1 were slightly but significantly bigger (Wilcoxon: p = 0.03) than the ones that were measured directly after sex sorting in the laboratory (2.09 mm vs 2.11 mm) and were significantly bigger than the recaptured laboratory males (1.96 mm) (Wilcoxon: p<0.001). There were no significant differences in size between mosquitoes recaptured within and beyond 25 m, 50 m or 100 m (Wilcoxon: p>0.05).

**Table 2:** Mean Distance Travelled (MDT) with Confidence Interval (CI) calculated by bootstrap, Flight Range 50 (FR 50), Flight Range 90 (FR 90) and wing measurement with standard deviation (SD) for OX513A-My1 and My1 laboratory strains.

Strain	MDT [CI]	FR 50 [CI]	FR 90 [CI]	Wing measurement [SD]
OX513A-My1	52.4 m [41.6; 61.4]	16.2 m [10.5;22.5]	142.3 m [116.5;157.6]	2.11 [0.08] (n = 223)
My1	99.8 m [79.6; 115.5]	59.7 m [42.8;73.6]	211.8 m [179.9;226.3]	1.96 [0.09] (n = 219)

95% confidence intervals were calculated by resampling individual mosquitoes with replacement and then reporting the 2.5 and 97.5 percentiles of the estimates obtained from 1,000 such bootstrap samples. n: sample size.

### Dispersal

Maximal dispersal was 220 m and 223 m downhill for OX513A-My1 and My1 males, respectively, and 96 m for both strains uphill. Six traps were set downhill at greater distances than these maximum recaptures but none uphill. Mean Distance Travelled (MDT), calculated with 25 m annuli, were 52.4 m and 99.8 m for OX513A-My1 and My1, respectively. MDT was significantly higher for My1 according to the bootstrap test whether trap 10 was included or not in the calculations. Flight Range 50 was 16.2 m and 59.7 m for OX513A-My1 and My1, respectively. Flight Range 90 was 142.3 m and 211.8 m for OX513A-My1 and My1, respectively. Both flight range estimates were significantly lower for OX513A-My1 males (Table 2).

Recaptures downhill were much higher than uphill recaptures even after trap density correction, suggesting that the released males flew further in this direction (Table 3). This non-random dispersal might be due to other factors such as wind, vegetation, topography, trap network, presence of field worker during the study, etc. There is insufficient data to speculate which factor or combination resulted in the observed dispersal.

**Table 3:** Downhill and uphill recaptures before and after correction for trap density [46].

		OX513A-My1	My1
Downhill	Original	2708	866
	Corrected	2409	770
Uphill	Original	315	46
	Corrected	437	64

### Survival

Last recaptures were made on day 9 and 12 for My1 and OX513A-My1 strain, respectively. Daily Survival Probability (DSP) ranged between 0.611 and 0.646 which were translated to life expectancy of 2.0 to 2.3 days after release for both strains whether calculated with or without trap 10, the closest trap which captured a disproportionate number of released males. There were no significant differences between the DSP of both strains (Table 4).

**Table 4:** Daily Survival Probability (DSP) and Life Expectancy (LE) with Confidence Interval (CI) calculated by bootstrap for OX513A-My1 and My1 strains.

Strain	DSP [CI]	LE [CI]
OX513A-My1	0.61 [0.569; 0.634]	2 days [1.8; 2.2]
My1	0.633 [0.592; 0.652]	2.2 days [1.9; 2.4]

95% confidence intervals were calculated by resampling individual mosquitoes with replacement and then reporting the 2.5 and 97.5 percentiles of the estimates obtained from 1,000 such bootstrap samples.

### Ovitrap monitoring

Ovitrap surveillance was conducted for 8 weeks from 20 December 2010 till 14 February 2011 in the uninhabited area (except for the week between 27 December and 2 January) and from 11 January 2011 to 14 February 2011 in the inhabited sites. No fluorescent larvae were found in either uninhabited or inhabited sites, indicating either that no mating occurred in the trial sites between OX513A-My1 males and wild *Ae. aegypti* females or that no inseminated females laid eggs in the ovitraps. This is unsurprising given the low number of available females in the uninhabited area and males, if any, likely to have travelled to adjacent villages where higher numbers of females were present. A large range of mosquito species were found throughout the ovitrap monitoring: *Aedes aegypti*, *Aedes albopictus*, *Aedes albolineatus*, *Aedes scutellaris* group, *Armigeres subalbatus*, *Culex sp.*, *Zeugnomyia gracilis*, *Tripteroides sp.* and *Toxorhynchites sp.* (Table 5). These findings are confirmed by the mosquito species caught in the adult traps; only one wild male and one wild female *Ae. aegypti* were caught during the study compared to almost 600 individuals of other species (Table 5).

**Table 5:** Number of specimens collected per trap per day during ovitrap surveillance and adult trapping by mosquito species.

Trap	Species	Uninhabited area	Inhabited area
	*Aedes aegypti*	0.02	0.15
	*Aedes albopictus*	9.82	16.70
	*Aedes scutellaris* group	0.26	0
Ovitrap	*Aedes albolineatus*	0.33	0.07
	*Armigeres subalbatus*	0.10	0.27
	*Zeugnomyia gracilis*	0.02	0.03
	*Tripteroides sp.*	0.04	0.25
	*Culex sp.*	0.15	0.05
	*Toxorhynchites sp.*	0.01	0.01
	*Aedes aegypti*	0.003	-
BG-Sentinel	*Aedes albopictus*	0.51	-
	*Armigeres subalbatus*	0.12	-
	*Culex spp*	0.22	-

**Figure 3:**
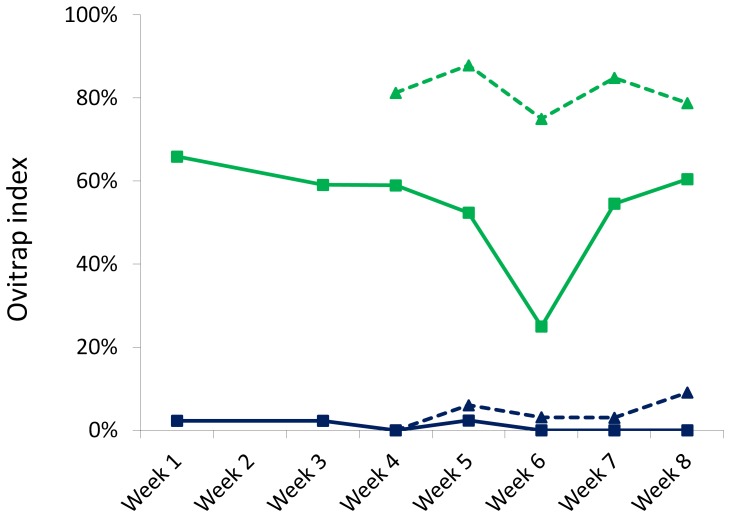
Ovitrap index of *Ae. aegypti* and *Ae. albopictus* in uninhabited and inhabited sites. The larval monitoring confirmed the predominance of *Ae. albopictus* in the uninhabited area as well as in the inhabited areas surrounding the study area. (*Ae. aegypti* uninhabited area: blue solid line; *Ae. aegypti* inhabited area: blue dotted line; *Ae. albopictus* uninhabited area: green solid line; *Ae albopictus* inhabited area: green dotted line).

The ovitrap index of *Ae. aegypti* and *Ae. albopictus* in the uninhabited area ranged from 0–2% and 30–66%, respectively, while in the inhabited area the ovitrap index for both *Ae. aegypti* and *Ae. albopictus* ranged from 0–9% and 75–88%, respectively (Figure 3). These results confirmed that *Ae. albopictus* was the dominant species in the uninhabited and inhabited areas.

### PCR

The PCR assay confirmed the fluorescence data. Of the 27 adults tested by PCR, 9 failed to amplify with the *Actin-4* primers indicating that the gDNA was of poor quality (probably due to degradation of the sample in the trap). The remaining 18 were all negative for the transgene, so were assumed to be wild individuals.

## Discussion

After release, the males have no access to tetracycline. For OX513A-My1 males this means that the tetracycline-repressible autoregulatory element is de-repressed [13]. Nonetheless, the two strains had similar post-release adult male longevity. Estimated daily survival probabilities were 0.61 and 0.63, equivalent to average life expectancy of 2.0 to 2.2 days, for the OX513A-My1 and My1 strains, respectively. These differences were not significant, so presence of the transgene does not appear to have a strong effect on post-release longevity.

A previous study comparing males with a 20% size difference found no difference in dispersal but increased longevity of larger males [49]. The two adult male cohorts used here differed in size by 8%. The lack of observed difference in survival may be due to the smaller size difference, statistical uncertainty or countervailing factors such as the presence of the transgene.

Daily survival probabilities (0.61 and 0.63) were within the range of those reported in previous mark-release-recapture studies using wild-type males: 0.53 to 0.85 [50], 0.57 and 0.70 [51], 0.41 and 0.56 [49], and 0.53 [52]. These previous studies were conducted in human-inhabited environments with significant wild *Ae. aegypti* populations. This indicates that the post-release environment of the present study, which was not typical of *Ae. aegypti* habitats [53] and was substantially free of *Ae. aegypti*, did not greatly affect male survival. However, relative to these previous studies the recapture rate was higher, i.e. 17.2% and 50.2% vs. 14.8% and 4.6% [51], 12.3% and 7.35% [49] or similar to studies where the males were released in houses which limits dispersal and enhances recapture, e.g. 23% [52] and 17% [54]. This may be a consequence of the lack of humans in the area. Despite not blood-feeding, adult males are thought to seek humans as part of their mating behaviour [55]. BG-Sentinel traps attempt to mimic humans and therefore compete for the attention of host-seeking females and males. In an uninhabited area the traps may therefore be much more efficient. This increases the statistical ability to compare the two types of male. The difference in recaptures between strains might indicate a differential attractiveness to the traps.

**Figure 4:**
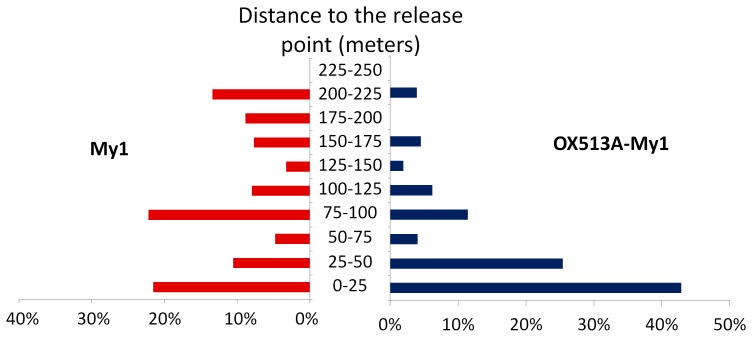
Difference in dispersal of My1 and OX513A-My1 males. Proportion of the total trap density corrected recaptures [46] calculated according to the distance from the release point by concentric annuli of 25 m. Most of the OX513A-My1 males (68%) were caught in the first two annuli surrounding the release point, i.e. <50 m, while the My1 males are more evenly distributed among the annuli (from 3% to 22%). This accounts for the latter higher Mean Distance Travelled (MDT), Flight Range 50 (FR50) and Flight Range 90 (FR90). (My1 males: red; OX513A-My1 males: blue).

The maximum distance of recapture (220 m and 223 m) was similar for both strains and higher than in studies conducted in human-inhabited environments in Thailand, 99 m [50]; Australia, 160 m [51]; and Brazil, 104 m and 132 m [49]. It was however lower than the 456 m maximum recapture reported in Thailand and Puerto Rico [54]. Mean Distance Travelled (MDT) for both strains (>50 m) was slightly higher than was reported in previous studies: 37 m [50], 35 m [51], 32 m and 42 m [49] and within the range reported by Harrington, 28–199 m [54]. This may indicate that males disperse somewhat further in the absence of females. Measured dispersal may have been affected by the unusually high recapture rate, leading to proximal traps acting as a partial barrier to dispersal; this would tend to reduce the estimated dispersal distance for both strains. High density of males in the cages before release might have induced dispersal [42]; however, the conditions in this respect were similar for both strains, validating the comparison of their dispersal. My1 males dispersed significantly further than OX513A-My1 males. It is possible that the presence or de-repression of the transgene is responsible for reduced dispersal of OX513A-My1 males; in flight mill experiments, OX513A males flew less far than wild type [56], however this may alternatively or additionally reflect differences in strain genetic background or unrecognised differences in pre-release rearing and handling. It is conceivable that the higher overall recapture rate of OX513A-My1 is due to a higher responsiveness to the traps, in which case proximal traps may have acted as a barrier to dispersal for this strain. Conversely, lower dispersal may have led to higher recapture rates, due to the higher density of traps close to the release point. The distribution of the proportion of corrected recaptures in each annulus tends to confirm this hypothesis. Captures of My1 males were evenly dispersed around the release point whereas OX513A-My1 males were mainly (68%) captured within 50 m of the release point (Figure 4). Consistent with a previous analysis [49], the size of the recaptured males was not correlated with dispersal for either strain.

More recaptures were made in traps downhill from the release site compared to those placed uphill. This correlation may not reflect causation – the non-uniform dispersal may be due to other environmental anisotropies such as wind, vegetation or surrounding human settlements.

It seems possible to conclude that males would not disperse further than 250 m and that OX513A-My1 has a comparable maximum dispersal but a lower median and mean dispersal than My1. Nonetheless, OX513A-My1 males show adequate dispersal capacity for their intended use in a sterile-male-release programme, though this should also be examined in an inhabited area.

As expected, very few *Ae. aegypti* were found by the ovitrap monitoring. Several other species were detected in the area but the recovered egg population was dominated by *Ae. albopictus*. This may not proportionately represent the adult species mix as the ovitraps used may be differentially attractive to different species of mosquito, however they have been shown to be effective for both *Ae. aegypti* and *Ae. albopictus* in Malaysia [44]. Ovitraps placed in nearby inhabited areas detected more *Ae. aegypti*, though *Ae. albopictus* still predominated. All aedine larvae were screened for fluorescence but none were found positive. No indication was therefore seen of any OX513A-My1 males reaching the village, as expected as this was far beyond the maximum distance travelled detected in this or previous studies. These negative data are also consistent with previous studies indicating strong mating barriers between *Ae. aegypti* and *Ae. albopictus*, and a complete inability to produce fertile hybrids if they are forced to do so in the laboratory [57].

The data from this study are encouraging for the potential future operational use of this strain and strategy in dengue control programmes. As with previous field releases, the transgene disappeared rapidly from the environment post-release, as expected, and was not detected more than a few hundred metres beyond the release area. Consistent with the prior risk assessment, no features were revealed that suggested any adverse effect on human health or the environment. The transgene seems to have little negative effect on lifespan; the apparent reduction in dispersal is not of a magnitude to prohibit operational use. The main caveat is that these observations should be repeated in an environment more typical for *Ae. aegypti* and where wild *Ae. aegypti* females are present. Given the highly urbanised, human-associated nature of this mosquito, that means an inhabited area.
